# Adversity in University: Cyberbullying and Its Impacts on Students, Faculty and Administrators

**DOI:** 10.3390/ijerph14080888

**Published:** 2017-08-08

**Authors:** Wanda Cassidy, Chantal Faucher, Margaret Jackson

**Affiliations:** 1 Faculty of Education, Simon Fraser University 250-13450-102 Avenue, Surrey, BC V3T 0A3, Canada; 2 Centre for Education, Law & Society, Simon Fraser University, 250-13450-102 Avenue, Surrey, BC V3T 0A3, Canada; cfaucher@sfu.ca; 3 School of Criminology, Simon Fraser University, 8888 University Drive, Burnaby, BC V5A 1S6, Canada; margarej@sfu.ca

**Keywords:** cyberbullying, post-secondary, impacts, health, well-being, safety, relationships, campus culture

## Abstract

This paper offers a qualitative thematic analysis of the impacts of cyberbullying on post-secondary students, faculty, and administrators from four participating Canadian universities. These findings were drawn from data obtained from online surveys of students and faculty, student focus groups, and semi-structured interviews with faculty members and university administrators. The key themes discussed include: negative affect, impacts on mental and physical health, perceptions of self, impacts regarding one’s personal and professional lives, concern for one’s safety, and the impact of authorities’ (non) response. Students reported primarily being cyberbullied by other students, while faculty were cyberbullied by both students and colleagues. Although students and faculty represent different age levels and statuses at the university, both groups reported similar impacts and similar frustrations at finding solutions, especially when their situations were reported to authorities. It is important that universities pay greater attention to developing effective research-based cyberbullying policies and to work towards fostering a more respectful online campus culture.

## 1. Introduction

Researchers over the past two decades have sought to understand and document the negative impacts of cyberbullying on youth [1]. Relatively little attention, however, has been paid to cyberbullying among adults. Some have suggested that that bullying and cyberbullying behaviors exist on a continuum from childhood into adulthood [2,3,4,5,6]. Cowie and Myers, for example, state that “bullying behavior appears to be embedded in a culture that … ‘normalizes’ such behavior and attitudes and shames bystanders into apathy” [4].

Information and communication technologies (ICT) permeate the post-secondary environment [7,8,9,10] and while its usage has brought many benefits, including improved communication and access to a wider range of learning materials and modalities, it has also resulted in a “rapid increase in uncivil online discourse” [11]. Email, for example, is a prime medium for cyberbullying [8,9,12,13,14].

Cyberbullying impacts Kindergarten to Grade 12 (K-12) students in the following ways: negative affect (e.g., low self-esteem, frustration, fear, anxiety), mental and physical health (e.g., depression, suicidal thoughts, headaches, sleep problems, stomach problems), academic performance (e.g., lower grades, absenteeism), school-related well-being (e.g., reduced concentration, isolation, feeling unsafe at school), and interpersonal relationships (e.g., alienation, trust issues, loss of friendships, exclusion, impacts on reputation) [15,16,17,18,19,20,21,22,23,24,25,26,27,28,29,30].

While comparatively less is known about the impacts of cyberbullying on university students and faculty, there are a few indicators. Pörhöla [6] demonstrates the long-term impacts of school bullying, which can persist into adulthood, and lead to trust issues, social isolation, anxiety, depression, withdrawal, and lower self-esteem. Recent studies on bullying and cyberbullying among university students report mental and physical health impacts such as anger, sadness, hurt, embarrassment, hostility, (social) anxiety, depression, suicidal thoughts, loneliness, relationship problems, decreased well-being, fear, crying, self-blame, lower self-esteem, poor concentration, low academic achievement, and absenteeism [2,8,31,32,33,34].

The limited research findings on cyberbullying towards and among university faculty members have not revealed as much in terms of impacts to date. The authors [8,9,35] note that cyberbullied faculty members report impacts related to productivity, confidence, concentration, relationships, mental and physical health, feelings of safety, and job satisfaction. The literature on workplace bullying and harassment in higher education indicates that bullied faculty are more likely to leave the organization, especially if they are untenured. Those who are tenured are more likely to stay; however, this can lead to an unhealthy workforce “at greater risk for negative health effects, absenteeism, and decreased productivity” [36]. Bullied faculty are less likely to feel loyalty to the organization and to use their voice to make positive contributions (see also [37]). Hollis’ [38] study on bullying in American universities indicates that a workplace victim spends 3.9 h per week avoiding the bully. She employs Hochschild’s concept of “emotional labour” to describe the work of maintaining “professional composure while withstanding emotional distress” [38].

This literature also describes victims’ feelings of powerlessness to stop the bullying and a reluctance to report the behavior for fear of not receiving future teaching appointments and of being perceived as incompetent to handle problems [13,39,40].

For more than two decades, bullying has been defined as a problem at least in part due to an imbalance of power [41]. Power imbalances are prevalent in the hierarchical context of universities: administrator to faculty member, senior to junior faculty, tenured to untenured, faculty to staff, supervisor to graduate student, instructor to student, not to mention imbalances based on gender, age, ethnicity, race, social status, and sexual orientation, which can permeate all of those relationships. The authors [8,9] have linked their analysis of cyberbullying at universities to the power and control model [42], borrowed from the field of intimate partner violence. This model incorporates tactics such as intimidation, threats, harmful language, social standing, exclusion, and harassment in the exercise of power and control over another in a relationship. ICT can be used to carry out any or all of these tactics.

It is also important, however, to recognize that the use of ICT may disrupt typical power hierarchies by providing the option of anonymity and enabling indirect forms of bullying [20,43,44,45]. DeSouza [44] notes that academic contrapower harassment is widespread in universities, meaning that a person with ostensibly less power bullies someone with ostensibly more power. McKay et al. describe how the university system is “weighted heavily in favour of the student, through the process of linking tenure and promotion to student evaluations” [13]. This disrupts the traditional hierarchy, where professors are perceived to wield power over students through grading [12].

Sallee and Diaz [46] found that lesbian, gay, bisexual and queer (LGBQ) faculty and staff are more likely to experience bullying in the university workplace from superiors, colleagues, and subordinates, highlighting the interplay of gender, race, ethnicity, and sexuality (intersectionality) when it comes to bullying. Lampman [39] found that women, minorities, younger faculty, those with less experience and credentials are more likely to be targets of bullying. According to Crookston [47], the majority of bullies are in higher ranked positions and use their rank, hierarchy, “cronyism” and “schmoozing” to cover up their nefarious behavior. Chairs who may have tendencies toward bullying behavior may be blind to such behavior in themselves or others.

In the emerging literature on cyberbullying in universities, the gendered nature of the problem has been highlighted [8,9,12,35,48]. In a survey of 331 faculty members from four Canadian universities, the authors [35] found that female faculty members and other teaching personnel were more likely to be on the receiving end of cyberbullying both from students and from colleagues. Female survey respondents expressed greater concern in general over this issue and those who were cyberbullied reported a wider range of negative impacts from it (see also [12]). The same authors also surveyed 1925 students at those four universities and found that, although cyberbullying victimization rates were comparable between male and female students, female students were much more likely to report a range of negative impacts affecting their personal and academic life [8,9]. Shariff and DeMartini [48] examine the prevalence of rape culture in university students’ online lives in order to better understanding the impact of misogyny on cyberbullying behaviors. They analyze several highly mediatized cases of gender-based cyberbullying and sexting, which can only be truly understood by examining their discriminatory and misogynist underpinnings.

The purpose of this paper is to examine the impacts of cyberbullying among adult populations in the context of post-secondary education. After describing the methods used in the study, the paper discusses the impacts reported by post-secondary students, faculty (including tenured and untenured professors, permanent and contractual instructors, student advisors, teaching assistants and tutor–markers—hereafter referred to as faculty), and administrators/policymakers (hereafter referred to as administrators). The paper concludes with suggestions and recommendations for policy and practice stemming from the research findings.

## 2. Materials and Methods

This paper offers a qualitative thematic analysis of various data sources relating to the impacts of cyberbullying on post-secondary students, faculty and administrators in order to supplement the authors’ quantitative findings, which have been reported elsewhere [8,9,35]. It adopts a grounded theory approach [49], whereby the themes were allowed to emerge from interaction with the data. Although prior awareness of the impacts of cyberbullying existed, through the researchers’ earlier work on cyberbullying in the K-12 (Kindergarten to Grade 12) sector [1,16,19], as well as their analysis of the aggregate data from surveys at the post-secondary level [5,8,9,35], the researchers did not allow this knowledge to constrain their interpretation of the presenting themes.

The data for this paper were drawn from several components of a larger study on cyberbullying at the post-secondary level, which included anonymous online surveys of undergraduate students and university faculty, in-person focus groups with undergraduate students, individual interviews with faculty members and university administrators at four participating Canadian universities from three provinces, as well as a policy scan of relevant policies at all Canadian universities. Results from various parts of the larger study have been reported elsewhere [5,8,9]. The research ethics board at the researchers’ university, as well as at the four participating universities, approved each part of the study. All participants gave their informed consent for inclusion before they participated in the study. The study was conducted in accordance with the Declaration of Helsinki, and the protocol was approved by the Office of Research Ethics at Simon Fraser University (#2012s0546 approved 31 July 2012). Pseudonyms were used for all in-person components (focus groups and interviews). Participants were assured that researchers would not disclose any identifying information.

In order to examine the impacts of cyberbullying, specific components of the broader study data were analyzed. First, one of the open-ended questions in the student survey and two of the open-ended questions in the faculty survey were used. These questions asked respondents to provide an example of when they were cyberbullied, how it made them feel, what they tried to do to stop it, and what happened (see exact wording of questions in Appendix A). In the student surveys (*n* = 1925), 145 participants responded to question #59. In the faculty surveys (*n* = 331), 28 responded to question #57 and 23 responded to question #92.

Second, the focus groups and interviews were audio-recorded and transcribed and then coded using NVivo software version 10.0.3 (QSR International, Burlington, MA, USA), according to themes that arose from the earlier analysis of the survey data [5,8,9], as well as new themes that emerged through a process of open coding [50,51]. In total, the researchers identified 39 references to the overarching theme of impacts in the student focus group transcripts (*n* = 10), 120 references to impacts in the faculty interview transcripts (*n* = 14), and 36 references to impacts in the administrator interview transcripts (*n* = 21).

Lastly, the researchers found that participants in the focus groups and interviews frequently employed vocabulary related to violence in their descriptions of cyberbullying. As such, a code was created to document the use of these terms, called “violence-based terminology.” During the coding process, the researchers captured this code under the category of the “nature of cyberbullying” while also referencing it in relation to the sub-theme of “concerns about safety.”

The coding process was iterative as the authors collaboratively established thematic codes based on their interaction with the data as well as their prior knowledge derived from the quantitative analysis of the survey data [8,9,35]. The second author then conducted an initial round of coding, which was verified by the first and third authors to ensure the presence of inter-rater reliability. During this verification process, additional themes were added to the initial scheme based on further analysis of the data. A second round of coding was then conducted, taking into account the clarifications made in the first round as well as the additional themes that had emerged. A third and final round of coding was conducted to ensure all themes had been captured and that the three researchers were in agreement.

## 3. Results and Discussion

The analysis of the various data sources related to the overarching theme of impacts led to the development of several sub-themes. These sub-themes were either reported as a result of: participants’ own experiences; indirectly through seeing a friend, colleague, or acquaintance experience cyberbullying; or as observed by participants as a result of their role at the university. The sub-themes highlighted in this paper reflect the dominant responses (based on the frequency and/or strength of the responses) within the broader thematic category of impacts [50,51]. The quotations selected for reporting below are exemplars, which reflect the dominant responses.

Although the data from students, faculty and administrators were analyzed separately, the researchers noted substantial overlap in the types of impacts raised by each group. As such, the data from students, faculty and administrators are discussed globally here, with an occasional reference made to specific distinctions that surfaced between the groups. The sub-themes discussed in this paper include negative affect, mental health impacts, physical health impacts, perceptions of self, impacts on one’s personal life and relationships outside the university, concern about one’s safety, impacts on one’s professional life, and authorities’ (non)response.

### 3.1. Negative Affect

The first sub-theme was negative affect; these references permeated all of the data sources. The negative emotions elicited by cyberbullying covered quite a range, from feeling sad, hurt, embarrassed, angry, humiliated, isolated, marginalized, and powerless, to wanting to retaliate and get revenge. Many talked about prolonged periods of crying, and being very upset, emotional, choked, crushed, wounded, and just feeling awful.

One faculty member, who endured years of cyberbullying by a colleague said:

I just collapsed. Like I remember I put my head down on my direct boss’ desk and I cried for, I don’t know, it felt like forever, probably just a few minutes but it felt, … I felt crazy.

(Debbie, 16 years’ experience, faculty interview)

A student who felt betrayed by a friend wrote: “The email taunted me, because I could re-read it…pass it to friends. It felt much more damaging and hurtful than words from her mouth” (Female 4th-year student, survey respondent S56).

Participants talked about feeling shaky, raw, irritable, and emotionally vulnerable. One student wrote: “I was worried other classmates would think I was a bad person because of what she was saying about me” (Female 4th-year student, survey respondent S915). Another student wrote:

I was cyber-bullied by someone that was anonymous, on a blog website … It made me feel very emotional and upset, because the website is public and can be viewed by anyone. This anonymous person had posted pictures of me and written comments below the pictures that were mean and not true.

(Female 3rd-year student, survey respondent S1334)

A professor described her experiences with a student:

Email, text messages making comments that I was incompetent, not accessible, was too slow, workload too difficult, and the words used were “useless”, “lousy”, and “I am reporting you to the (professional association), they will take away your license you are so stupid”. Student was not open to feedback. I felt attacked, humiliated, and believed all the other classmates were feeling the same way, as it was veiled in the notes that everyone thought I was a ‘loser’ instructor.

(Female professor, 6–10 years’ experience, survey respondent F252)

A teaching assistant and instructor described her reaction to being cyberbullied by a student: “I was very taken aback by the unfair accusations in the e-mail. I was shocked, then angry” (Female, less than 2 years’ experience, survey respondent F167).

Several faculty members were upset with the anonymous messages from students posted on external professor-rating websites and frustrated by the fact that they were not able to get these removed by site administrators. As one professor stated:

I know there are cruel and disparaging remarks posted about me on ratemyprofessor.com … The unpoliced internet allows students to post demeaning and often untrue comments anonymously. How is this not libel?

(Female professor, 2–5 years’ experience, survey respondent F63)

Although a few respondents mentioned retaliation or revenge, it was more common for them to say that it left them feeling powerless in the situation. Some said they did not know how to respond, while others said that responding made things worse.

I was sent lots of text messages from an individual who believed I had been gossiping about her. She was threatening, and told me to fix the problem I had caused. She texted me 73 times in one day, and over a week it was about 180 messages. When I didn’t respond it was worse.

(Female teaching assistant, less than 2 years’ experience, survey respondent F42)

One professor noted that he was: “Unfairly treated with no chance to defend myself” (Male professor, 2–5 years’ experience, survey respondent F260) and another simply stated: “I felt powerless” about being cyberbullied by students (Female professor, more than 10 years’ experience, survey respondent F283). Several pre-tenured professors commented about being cyberbullied by senior colleagues.

### 3.2. Mental Health Impacts

In addition to the negative affect brought on by the experiences of cyberbullying, some faculty and student participants discussed more generalized mental health impacts, including stress, anxiety, depression, and suicidal thoughts. A common response was that the situation was “stressful” or “hard to deal with” or that they felt “stressed out” or “exhausted” by the situation. One faculty interviewee described it as “carrying that weight with them” (Claire, teaching assistant/tutor–marker). One student wrote about a “vicious” reply from a male professor to her request for clarification about some of her grades: “Eventually, I did message him with clarification about my first message, and arranged an in-person meeting, but it had still caused me a massive amount of stress and personal suffering” (Female 1st-year student, survey participant S3). Another student described being attacked, impersonated, and threatened by an anonymous person in an online forum. The student eventually changed his email and all online accounts. He wrote:

The effort was a lot, but as a result I managed to dissociate the old from the new, and no further harassment continued. No crowds, forum members targeted me nor was my identity compromised, so I was lucky. Despite that, the fears of being attacked in such a manner and knowing the true realistic sense of what could possibly have happened was unnerving and stressful enough for me that time that it affected me quite a lot that time.

(Male 4th-year student, survey respondent S941)

Participants also discussed feeling anxious and worried about re-occurrence. One professor, for example, talked about negative email messages being circulated throughout her department, where senior faculty members would belittle and intimidate junior faculty members to get their way. Responding to those emails consumes a lot of time and mental energy as well as generating anxiety about the next message that will come back, as noted by this respondent:

Yeah, the next wave to come and hit you. And that’s another emotional, like the anxiety of that starts to build and it’s like “Oh, it’s here. It’s gonna come again.” And every time you turn on the computer, it’s: “So, is there another email that’s gonna come?” So it’s the anxiety of that builds.

(Skylar, female associate professor, 7 years’ experience, interview)

Both students and faculty members mentioned panic attacks brought on by the cyberbullying situation. One student wrote about a cyberbullying incident at the hands of her so-called friends: “I remember experiencing my first ever panic attack, and several more came that day. … I later developed depression because of this. It was one of the most darkest moments in my life” (Female 4th-year student, survey respondent S978).

For others, the stress and distress of the cyberbullying led to depression and, in some cases, to suicidal thoughts.

The bully wrote a status telling me I should kill myself and that I was ugly. It affects my mental health, I felt very depressed.

(Female 1st-year student, survey respondent S1144)

A friend called me fat and told me i should just forget about finding anyone to love me. This made me feel really sad and suicidal, but I just tried to ignore it and continue with school and eventually I recovered by the help of my true friends who I have known for some years.

(Female 4th-year student, survey respondent S47)

... But those DID make me feel suicidal, depressed, like it was ruining my friendships inside and outside of school.

(Male 1st-year student, survey respondent S1556)

Multiple respondents sought counselling to address their depression and suicidal thoughts stemming from their cyberbullying experiences. One faculty respondent, who had been serving her university as an administrator, described falling into a deep depression and requiring 18 months of counselling due to a campaign by a group of colleagues to have her removed from her administrator role. She wrote: “I doubt that I will ever fully regain my confidence: my survival mode now in another academic unit is to remain ‘under the radar’ of all administrative personnel and most of my colleagues” (Female professor/administrator, 6–10 years’ experience, survey respondent F161).

### 3.3. Physical Health Impacts

Physical health impacts were another category of impacts discussed by participants. These impacts, sometimes referred to as “physical signs of stress,” specifically included sleep problems, stomach problems, and weight loss.

One student described an incident where alleged friends pretended to be her “crush” online, engaging in a lengthy conversation during which they targeted her insecurities and then revealed themselves and laughed at her some more. She wrote: “I felt deceived in so many ways and sick to my stomach” (Female 4th-year student, survey respondent S978). One faculty member discussed her stomach being “in knots” for departmental meetings due to cyberbullying as well as face-to-face bullying she had been experiencing from a group of colleagues (Lynne, female adjunct professor, 8 years’ experience, interview).

Another student wrote about cyberbullying by a former intimate partner:

When I finally stood up for myself, he threatened me almost every day, sending fake e-mails and he even tried to impersonate me and tell the police that I was sending him and his family nasty e-mails and phone calls … I had panic attacks every day and I lost a significant amount of weight. I still bear the scars that he left me, physically and emotionally.

(Female Master’s student, survey respondent S1358)

One administrator described an incident where a faculty member’s complaint had not been handled adequately by their university.

If the Dean had acted on the request of the faculty member a while ago, he would be in a much better mind set right now. And I’ve let that be known because there’s more complications that have come out of that. And it’s had a very negative effect on this health.

(10494, male faculty association representative, administrator interview)

Further, as one professor explained, sometimes there are mechanisms within the university that inadvertently enable cyberbullying and its damaging effects to occur:

Sometimes I feel like you have to justify why you’re getting these course evaluations … I say my agenda is equity in human rights and I’ll stand behind that every time … But again students … they resist that challenge and so feel that their only mechanism is to … take it out on you on the evaluation where they get their one shot. So I just think they’re antiquated and … can be quite damaging, but nobody looks at them that way which can be a problem.

(Jeff, assistant professor, 3 years’ experience)

### 3.4. Perceptions of Self

The fourth sub-theme in terms of the impacts of cyberbullying pertains to perceptions of self. Participants indicated in several instances that the cyberbullying they experienced affected their self-confidence, self-esteem, and/or self-image. While a few commented that they started blaming themselves for what had happened, the more prevalent response was that they began to believe what the cyberbullies said about them and developed inadequacies as a result. This impacted their personal and professional lives. As one student wrote:

I am a male and was placed in work team, which included another female student. Several messages were sent to me by this student, which made me feel as though I am inept at interacting with women. It was also implied by her communications to me that there is something pathological about being a 22-year-old male who has never had a girlfriend. Overall, these communications made my confidence in interacting with women regress significantly, something I have struggled with since my teens.

(Male 4th-year student, survey respondent S952)

In another example, a teaching assistant’s confidence in her work was called into question when she was cyberbullied by a student:

Student was rude and demeaning regarding an issue in the course for failure to hand in an assignment and thus subsequent grade. I felt like my marking style was put into question as well as my confidence as a teaching assistant/marker.

(Female teaching assistant, 2–5 years’ experience, survey respondent F12)

In another instance, it was the tutor–marker’s inappropriate online comments that affected the students’ self-confidence and self-esteem:

The Tutor-marker for our class was repeatedly rude in her comments to students in the online class, and I felt like some of her comments were demeaning. They weren’t always directed at me, but rather at groups of us who were asking honest questions. Her responses made people feel dumb/ignorant/as though they were bothersome and made people not want to take an online class again. In talking to classmates, several felt unsupported and as though they should not ask for help or clarification when they needed it because they were afraid of how she would respond or how she might judge them/grade their work differently.

(Female 4th-year student, survey respondent S90)

Further, some students reported that their self-image had been affected by the cyberbullying they experienced. For instance, one Aboriginal student, who did not supply many details, simply wrote: “I felt inadequate and I questioned my Status as an Aboriginal” (Female 3rd-year student, survey respondent S196). Another student wrote:

My ex-boyfriend said hurtful things to me against my personality and the way I look. Even though I asked him to stop he would not stop ... at one point i started to believe everything he said and I felt worthless and my self esteem was pretty low.

(Female 1st-year student, survey respondent S1449)

As one focus group participant explained, it can be difficult to move past negative comments, even when you want to believe they are not true. The repetitive aspect of seeing the message time and time again on the internet serves to reinforce this interpretation.

… you don’t have to listen, but it’s so easy, you know, once you’ve seen a comment, a bad comment, even if you know it’s wrong … you can’t unsee that. And even if it gets taken down in a day, you will, if you saw it, it will still eat away at your soul kind of thing.

(Wallace, male 1st-year student, focus group participant)

### 3.5. Impacts on Personal Life, Home Life and Relationships Outside the University

Negative effects were also felt in participants’ personal lives, outside of the university context. Several discussed avoiding certain people, places, social media sites, and activities as a result of being cyberbullied, as well as its influence on their relationships. This avoidance in described in these two examples:

I have deleted all contact I have with (the cyberbully) and avoid him at all costs. I have had to drop several other extra-curricular activities because he was involved in them.

(Female 3rd-year student, survey respondent S286)

I was angry with myself for believing she was a good person, for being vulnerable and inadvertently giving her ammo with which to attack me. I was upset because a friend of hers wanted to date me and sent me a harassing text at her behest, which was extremely disappointing. … Sometimes when I go to the food bank or Women’s Center, I worry I’ll see her there.

(Female 4th-year student, survey respondent S915)

In several instances, participants reported being bullied by someone whom they had thought was a friend; this led to trust issues and sometimes a severed relationship. As one student wrote: “Since no one was helping me I decided to remove/block all the people who were bothering me on FB and stopped talking to them in real life” (Female 2nd-year student, survey respondent S1230). Another student wrote: “Told her I don’t want to be friends and to leave me alone” (Female 3rd-year student, survey respondent S510). Another student who had been cyberbullied by a former friend and roommate described the aftermath as follows: “I accepted her apology and we can say hi and chat, but I will not seek her out as a friend” (Female 4th-year student, survey respondent S1878).

Participants reported impacts that affected their wider relationships, not only with those who cyberbullied them. As one student wrote:

A student at another university sent me really rude, hurtful messages. I felt really bad about myself and it really has ruined not just that friendship but almost all of my friendships. To stop it I just removed myself from that group of friends.

(Female 2nd-year student, survey respondent S659)

Another student described her feelings of distrust of friends that became generalized.

I was very upset, unsure of why this person decided to select ME to do this to. This incident made me feel embarrassed, upset, and isolated me from others because I did not know WHO had posted that about me, so you start to question who your friends/enemies are.

(Female 4th-year student, survey respondent S1398)

A few talked about the impact on their marriage and partners, as indicated by these two faculty examples:

I think it impacted my marriage to some extent. I was in a horrible mood and state all the time.

(Lynne, adjunct professor, 8 years’ experience, interview)

I remember feeling like I haven’t felt for years and years and years and years, just really shaky in myself, really raw, shaky, everything being magnified, any comment my husband made being magnified.

(Debbie, faculty member, 16 years’ experience, interview)

### 3.6. Concern about One’s Safety

Several respondents explained that the cyberbullying they had experienced or witnessed was fear-inducing and evoked concern for their personal safety or that of others. This part of the data set, from each set of respondents (students, faculty and administrators) was imbued with terminology related to violence. The student focus groups, faculty interviews, and administrator interviews were coded in NVivo for the use of “violence-based vocabulary.” The student focus groups included 94 references to such terms, the faculty interviews 101 references, and the administrator interviews 69 such references.

A gamut of terms referring to interpersonal violence and aggression were used, such as: abuse, aggressive, assault, attack, bashing, beat down, danger, dead animals, defend, enraging, fight, force, harassment, hate, hit, horrific, hurt, intimidation, jostling, kill, lashing out, mob mentality, phone screaming and roaring and shouting, poison, power and control over someone, push/push back, retaliation, risk, road rage, seized upon, stab, stalker, swarmed, threats/threatening, victimize, vindictive, violence/violent pictures, and wounding. Further, a number of words invoked the imagery of weapons, such as: backstabbing, firing back, shield, stabs you, sword, take a knife out, target, and weapon. In addition, the vocabulary of war also permeated the discussions: battle/battleground, blast off an email, bombardment, colonial attitude, combat, conflict, create campaigns and targeting against you, cyber war, defend, a host of disaster, and window warriors.

Not only did participants use these terms in relation to the fear they were experiencing, several also expressed concern that the cyberbullying would not stop. They gave examples of dreading turning on their computer or accessing a particular application, such as the course-based WebCT, where they did not know what the next wave of abuse would bring or when it would come. In other instances, they were worried about coming face-to-face with the bully:

A colleague was not happy about the views of committee members and used email (and) in-person aggression to influence committee members. This included hovering over me at my desk to persuade me towards her position. I wanted to run when I saw her in the corridor as her aggression was very overbearing.

(Female professor, 6–10 years’ experience, survey respondent F251)

In another example, it was the spread of the cyberbullying to one’s friends that caused greater concern:

I was recently photoshopped into another photo - my face onto another person’s body, and it was a shocking experience because I had assumed the bullies had left my friends and I alone. … I felt personally attacked, as well as feeling scared that they would do the same to my friends as well.

(Female 1st-year student, survey respondent S611)

Respondents also expressed fear that there would be an escalation from online/verbal attacks to physical attacks.

Over Facebook I was sent a personal message from a bully. Disclosed within this message was a death threat. They threatened that if I didn’t stop coming to school and being who I was that they were going to kill me. It was the scariest time of my entire life.

(Female 1st-year student, survey respondent S1534)

Over the past week a male student from a seminar class has sent me several demanding, angry, and belittling emails (essentially he is upset about quiz grades and that he’s past the drop deadline from class) ... I feel strongly that such behavior would not occur were I older, and/or male. For the most part I accept this as an unpleasant part of my job, but it certainly makes me feel unsafe working in the evenings in my office when no other faculty members are around. (I don’t know what an angry student is capable of and I don’t want to find out.)

(Female professor, 2–5 years’ experience, survey respondent F63)

What emerged was a real targeting of one of my staff members and the comments went beyond the event and got quite personal and then definitely bordered on threatening. … he was pretty bothered by it because … he actually lived in residence and so lived in a quite high profile position and so he contacted protective services to have them look into it a potential violation of the code.

(Katherine, residence director, administrator interview)

One faculty member who researches sexual minorities, and who is targeted regularly for his views, said that he tries not to let the cyberbullying “debilitate” him or “mobilize [him] to live life in fear.” He prefers to use the anger he receives in a “productive way” to drive positive change. However he also noted that

it makes me, you know, mindful … as a gay man … I’m mindful of my surroundings … where will the next attack come. I’m always aware on the street who’s walking behind me, who’s ahead of me, where I am and, well, even the person beside me.

(Jeff, assistant professor, 3rd year at the university, interview)

Another faculty member and administrator, who works in the area of violence against women and has a strong media presence, described how she repeatedly has been threatened online for her work. While she tries not to let it affect her personally or take it too seriously, she acknowledged that: “I suppose, you know, these things are worrying in an objective way.” She described one incident:

It had an impact on my department because, you know, the support staff especially were quite concerned about the potential for someone to come into … the building and to be disruptive or worse, so for a time our (administrative professional officer) made a decision to lock our door so that, yeah, so it did have an impact on my unit.

(Jane, professor and chair, 17 years at the university, interview)

### 3.7. Impact on Professional Life

This sub-theme encompasses those impacts on faculty members’ work lives and on students’ academic studies. Many of the impacts discussed above also may directly or indirectly impact “professional life;” however, the impacts discussed here are those specifically addressed by participants.

The first example, reported by both students and faculty, concerns the avoidance of, or lack of full engagement, in various online aspects of university life. Although online content management systems are now commonplace in the post-secondary sector, several respondents noted the dread they felt when opening certain applications. For example, one student described a situation in which she was berated by her professor in a message posted to the class board on WebCT. She described the incident as “humiliating and stressful” and stated: “I have since opted to NEVER post on webct unless required to do so for participation” (Female, 4th-year student, survey respondent S501). Several professors and administrators said that they now refuse to engage with others over email because of their negative experiences with email, although some commented that this may be a positive move since it is obviously better to engage with people face-to-face rather than risk being misinterpreted over email.

Participants also discussed how cyberbullying had impacted their relationships with others at the university. Several faculty members who had been cyberbullied by students commented that they now had a generalized distrust of all students, as noted here: “It also has caused me to be more strict about rules with students and more standoffish in general” (Female professor, 2–5 years’ experience, survey respondent F63). An administrator who commented on the experiences of faculty members who had been cyberbullied by students stated:

I think it changes a faculty member’s approach. … I know it gets them second guessing on what they do, on how vulnerable they make themselves, on decisions that they make because they don’t want to go through that again. So it kind of changes the relationship between the faculty member and the student.

(10494, male faculty association representative, administrator interview)

The inability to concentrate was noted above under the category of mental health impacts; this impacted the victim’s ability to work and to work well. Students shared that they became uncomfortable in class or when asking for help, and this impacted their grades. Professors described not living up to their own or others’ expectations. One professor, for example, stated that successive waves of bullying emails drained her energy and influenced her decision not to respond because it was not worth the effort and stress of psyching herself up to defend her position. She stated, however:

If I’ve decided to disengage, then there’s the guilt of that, you know, I let the bully win and it also makes me withdraw from my position, it makes me withdrawn from my professional position as a member of the community, it makes me feel like it’s really not worth it to, for instance, deal with substantial problems and how we run certain programs.

(Skylar, female associate professor, 7 years’ experience, interview)

In another example, a faculty survey respondent described a situation where she believed she had been deliberately excluded from exchanges about a group project by a senior colleague bully due to her past criticism of decisions taken by the bully. When the situation came to light and the bully was confronted, the bully feigned ignorance and offered an apology. However, this apology was never shared with the victim’s colleagues, so others were not aware of why she had been absent. The respondent commented: “her bullying and her apology occurred in the privacy of email. So my colleagues, at least some, must have believed I was unprofessional if not unresponsive” (Female professor, 6–10 years’ experience, survey respondent F27).

A related issue is the financial and career impacts discussed by those who experienced cyberbullying. One TA/sessional instructor reported that she felt she had to pretend nothing had happened, as she was afraid she would lose her job if she said anything (Michelle, faculty interview). Some reported that they had lost certain teaching assignments, or that their classes wouldn’t fill due to certain comments on professor-rating websites that could not be taken down. One teaching assistant commented on a situation involving her husband who was a first-time sessional instructor when he became a cyberbullying target. He went from being optimistic to being hurt and jaded. His career aspirations changed; he decided that he longer wished to become a professor.

The ‘publicness’ and reach of cyberbullying was also mentioned as factors. As one administrator commented:

The impact of the Internet is materially different from me writing a sign on the wall which says (interviewer’s name) is a slut. There’s only a finite number of people who are gonna walk by and see that sign … So we need to sit down and very carefully work through, do we need something that can respond at a different level, given the impact, the power of the Internet?

(Charles, senior academic administrator, interview)

Defamatory comments on professor-rating sites remain in place for all future students to see. Belittling or humiliating emails that are copied to entire departments or list-serves also have a broad impact. Several participants described such incidents within the context of power mongering, particularly with senior faculty members putting down junior faculty members. Several professors also commented on having their tenure or promotion threatened by the bullies.

In some cases, students and faculty left the university because of their cyberbullying experiences. Of course, those who had left were not in a position to participate in the study, although second hand reports were provided by other students, faculty members and administrators. For example, one faculty member, who had also been serving as an administrator, had been involved in the case of a student who had been cyberbullied by individuals living in the same university residence:

And this is how I got word of it because she said: “I don’t want this (scholarship) money to be used for the residence since I have to move out, I cannot psychologically, I’m not able to stay in there anymore.” And this was when I got involved and wrote the letter in fact to the residences saying these are the reasons she had to leave…you have confirmation of the physician... And what was interesting…the student in the end decided to leave, what I regret is the fact that the student didn’t feel comfortable in staying at (University A) and that she was forced to leave because of this experience.

(Claudia, assistant professor and centre director, 2 years in current position, interview)

One faculty member described a group of colleagues and staff at her former university who would bully others and attack anyone who went against them, leading to her departure from that university, along with several others. She said:

I mean they just kept having people leaving and leaving and leaving, but there was no ah-ha moment, maybe it has to do with something, it was always the blaming and I mean I heard through the grapevine that they were really angry with me after because I left in the middle of the year … but I couldn’t stand it anymore, so I quit. …

It just has such a detrimental effect … on a person’s life, on a person’s home life, on their job satisfaction. I mean the reason that I didn’t leave long before I did is I actually enjoyed what I was doing, the actual job itself. I loved my students, I loved the program, I enjoyed teaching the courses … I was absolutely committed to the students and to the program, so it was really hard to leave.

(Lynne, adjunct professor, 8 years’ experience, interview)

Others discussed impacts on the department as a whole. These impacts are discussed below in the context of administrators’ inadequate response to complaints and the perception that bullies can get away with it, leading to others disengaging to avoid being (further) targeted.

### 3.8. Authorities’ (Non) Response

The final sub-theme that emerged related to participants’ experiences with the responses they received from the authorities they went to with their complaints. Authorities included departmental administrators, senior university personnel, or authorities outside the university such as the police or website administrators. Participants noted the ways in which the response (or lack thereof) from authorities had the potential to make a huge difference, both in positive or negative terms.

While participants overwhelmingly focused on the lack of adequate or appropriate responses, those who did receive help were generally appreciative of the support regardless of whether the outcome was ideal. For instance, one student who received a prejudiced comment from a classmate on a class website wrote:

I informed the professor, she dealt with it. The student gave me an apology in class, and remained silent for the remainder of the term with regards to his prejudice comments. … I was grateful for the professor’s support and for the public apology.

(Female 4th-year student, survey respondent S1223)

In other examples, a sessional instructor and a professor each were glad for the support they received:

The student sent me a rude and derogatory email. I was shocked and upset as I work hard to have positive and open relationships with my students. I informed my immediate supervisor and we booked a private meeting with the student. Although we reviewed the inappropriateness of the behaviour with the student, I don’t really think she understood (or cared) about what she had done. I was happy that we had the meeting with her but don’t think she understood the gravity of her behaviour.

(Female sessional instructor, 2–5 years’ experience, survey respondent F152)

A colleague sent me a series of insulting and demanding emails—she also copied approx 10 other colleagues on these messages. I immediately informed my Chair, the Union, and the Human Rights Officer, all of whom were very supportive, and all of whom met separately with the individual. While it was a very unpleasant experience, I did feel like several people intervened on my behalf and the harassment stopped.

(Female professor, 2–5 years’ experience, survey respondent F309)

The administrators who participated in the study generally had a greater awareness of the situations that do get handled appropriately at the university level. For instance, one administrator, who had only been peripherally involved in a case where a faculty member had been receiving threatening emails from an unknown email address, commented:

I think there are lots of policies and procedures in place, there’s lots of offices that work together …

That was pretty impressive how quickly offices and individuals work together to coordinate I suppose a response to that in a very timely way. So it, the system worked and actually that’s been my experience with these sorts of things.

(Jimmy, IT Services, administrator interview)

Respondents at all levels noted that if a concern was raised to an authority, it was important to feel supported:

…when the issue (student cyberbullying a faculty member) was raised from the faculty member to the Dean, there was instant support and it was nipped in the bud right away. And, you know, if it wasn’t, it would have just kept going on, that student would have been allowed and they would have been supported by no action and it would have just gotten bigger and bigger and bigger and bigger … I’ve had instances before where I had support from the Dean and it made all the difference in the world when I was instructing, you know. And having that confidence of an administrator supporting you, not necessarily acknowledging that you were right, but supporting you or working those things through is huge.

(Janet, senior academic administrator, interview)

In addition to providing support to faculty members confronting such situations, a well-handled intervention also can have the effect of educating the student(s) involved. As one administrator commented:

And it was an educational moment for the student …to say look it, you can’t be doing this because of X, Y, and Z, this isn’t gonna achieve what you’re trying to achieve.

(10494, male faculty association representative, administrator interview)

Despite the somewhat positive experiences noted above, negative experiences were more typically the ones participants recalled. At one of the universities, two of the interviewees worked in the same department and had both been targeted by the same colleague over a prolonged period of time. Both expressed a great deal of disappointment in the lack of support they received from their university, particularly because they were not the only targets in their department, and although some interventions were attempted with this individual, none were successful.

We had a “lengthy conflict resolution process” (in air quotes for the tape) with a conflict resolution specialist. We had about six sessions and apparently that made things worse in the long run. … if you think of the parallel of marriage counselling, you can’t do marriage counselling in a situation where one partner is still actively abusing the other, right. That has to stop before you can say “are we gonna salvage this?” …

And then … we did all kinds of things about … developing group norms and things like that which were … ineffective at best and harmful at worst. One of the recommendations that this conflict resolution expert made was that [name of bully/cyberbully] should undergo some anger management training … he perhaps maybe did go off and have some sessions, but this then became … a weapon for him to use in future online bullying where he says, you know, four (colleagues) tried to get me fired and I had to go for anger management counselling and it’s now another thing for him to be bitter and angry about, etc.

(Debbie, faculty member, 16 years’ experience, interview)

Despite some efforts at conflict resolution in this case, both respondents commented that they generally felt unsupported by their university and that the university “did nothing.” This lack of response on the part of the university was what allowed the bullying and cyberbullying they experienced to continue for years. Others also commented on the lack of response by their universities and how it left them feeling unsupported and alone to confront their problem.

Participants also noted that the lack of response from the university allowed the same problem to reoccur. One professor gave an account of a student who relentlessly tried to get their grade changed by begging, making false statements, threatening, and making nasty comments about the professor on the course’s student chat site. The professor felt unsupported by the administrator who was relatively inexperienced and changed the grade to prevent the situation from escalating. She concluded: “Unfortunately this likely made her feel like she had ‘won’, possibly fueling the same behaviour in the future” (Female professor, more than 10 years’ experience, survey respondent F26).

Another female professor discussed the lack of response from administrators towards a senior colleague who had bullied her. This individual used his position of power to demand a grade change, storming into the office to confront her, shouting at her at a public event, and writing nasty things about her during her tenure case. She noted: “the worst thing was that nothing was done. This person had no repercussions and this shows that bullies can get their way” (Female professor, 6–10 years’ experience, survey respondent F174).

Students also described feeling powerless when no one at the university would take action on their complaints of abuse:

I just felt that one of my TA’s was threatening, and talking inappropriately to students in a condescending way. It made me angry, and I felt that because I am paying for this I deserve someone to be respectful … Another TA I had verbally and emotionally abused many classes. Numerous students contacted the department in hopes that something would be done but nothing was. It was highly unprofessional and appropriate and very discouraging for such a good university.

(Female 4th-year student, survey respondent S552)

Faculty also shared their frustrations with web site administrators who refused to take down false, defamatory, and abusive comments, leaving participants feeling powerless. As one faculty member noted:

I asked ratemyprofessor to remove the category called “hotness” marked by a chili pepper icon from my profile, explaining that it reduced my dignity, was defamatory, and implied that my appearance affected my teaching, and could even imply that I would use “hotness”, i.e., flirting or sexuality in the classroom to get my way (against all university policy). Again, this undermines my dignity, is defamatory, and humiliating. ratemyprofessor neither responded nor removed the hotness rating. This is a company in the US. I am not pursuing it further.

(Limited-term lecturer, gender identified as “alternative,” 2–5 years’ experience, survey respondent F233)

Power mongering, manipulation, and exploitation of well-connected relationships were noted as ways that people who engaged in cyberbullying managed to escape the consequences for their behavior. Multiple examples were provided by participants of (usually) senior colleagues banding together to undermine their target in the eyes of colleagues or upper administration. In one instance, a female professor became a departmental administrator and attempted to put an end to disrespectful behavior that had been occurring in her department, where junior faculty members were told not to speak at meetings and were generally bullied by senior members. The bully group then turned on her and managed to have her relieved of her administrative duties, with the help of their friendship with the Dean, at which point one of the bullies sent a message stating: “ding dong the witch is dead.” She stated: “I have never experienced such intense hatred of others and perverse power manipulation” (Female professor, 6–10 years’ experience, survey respondent F161). A faculty member at another university described the power mongering and manipulation as follows:

I think in the university it’s still very hierarchical and I think sometimes different groups may get together and they kind of are hiding behind academic reasons for their abusive positions but yet that’s still what they’re doing is abuse.

(Molly, faculty member, 10 years at university, interview)

Faculty members who had experienced or observed bullying or cyberbullying discussed the toxic work environment that is created when no action is taken to rectify the situation, and their problems are either ignored or swept under the rug. Several participants talked about adopting a defensive posture, avoiding email conversations, meetings, or other collaborations, which might invite further abuse. One faculty interviewee, who had been the target (along with some of her colleagues) of bullying and cyberbullying by one colleague in her department, commented that, “The energy in our department is completely frozen.” As a result, she and others had decided:

Not wanting to pop our heads … not wanting to have any kind of new initiatives in the department because then it’s, you become a target … right, so if you say “hey I’d like to do this, what does everyone think?” you know, then that makes you visible. …

I’ve made that a very conscious strategy, focus on what I love which is … working with my students and I have control over that and I can do a great job and grow and challenge myself and just completely detach from any bigger departmental initiatives, plans, strategic planning or anything like that.

(Debbie, faculty member, 16 years at university, interview)

It is clear that individual departments and universities as a whole lose out from not addressing bullying and cyberbullying behaviors adequately. Students become disillusioned, some leave; faculty members disengage, do not provide all that they have to offer, and some of them leave the university as well, all of which imposes a greater burden on administrators who are left to pick up the pieces once situations have become out of hand.

## 4. Conclusions

The analysis of open-ended responses to select questions from the student and faculty surveys, the student focus group responses, and the faculty and administrator interviews revealed that the victims of cyberbullying at the four Canadian universities studied experienced a myriad of serious, negative impacts. Students reported being targeted primarily by other students, while faculty members, whether junior or senior, tenured or untenured, or in administrative roles, were targeted by both students and colleagues. Interestingly, both students and faculty reported very similar impacts to being cyberbullied. Both groups highlighted their negative affect; for example, feeling sad, embarrassed, hurt, humiliated, wounded, marginalized, with some wanting revenge and to retaliate. Violence-related words were also used when describing their experiences and frustrations with trying to resolve their situations: hate, kill, abuse, attack, assault, hit, stalk, stab, roaring, bashing, lashing out, weapon, just to mention a few descriptors. Several respondents reported feeling unsafe, psychologically as well as physically.

Both students and faculty described mental and physical impacts such as depression, anxiety, stress, sleeplessness, stomach aches, weight loss, and even suicidal thoughts. Their experiences being on the receiving end of cyberbullying affected their work lives and their personal lives. Students reported that it affected their grades and relationships inside and outside the university, including avoidance of certain individuals and places where the cyberbully frequented. Faculty members also reported trying to avoid colleagues who had cyberbullied them, with several saying that they wanted to quit their jobs, with some actually changing campuses to avoid the bully. Respondents’ self-concepts and sense of self-worth were also negatively affected.

Of the students and faculty who reported their concerns to superiors, administrators, or other authorities, only a few expressed a degree of satisfaction with the response and/or the outcome. Most expressed frustration with the inadequate responses they received, with the issues they raised either being ignored, buried, or deflected with few solutions offered. Faculty members commented that if they did complain, that they risked being seen as the problem and that they felt this impacted their reputation as well as their ability to advance. They generally felt alone and isolated, whereas they saw the culprits thriving in a culture that was rife with power mongering, manipulation, and exploitation of well-connected relationships.

None of the administrators who were interviewed had experienced cyberbullying themselves (or at least they did not report that they had), although some of the faculty respondents also occupied administrator roles and did report being targets of cyberbullying. A few of the administrators had been asked to address issues of cyberbullying that had come to their attention and they generally they felt that these situations were adequately resolved. They noted the importance of relevant policies that are accessible to students and staff, and for immediate supervisors (such as chairs and deans) to effectively address the problems when they first surface, so that the issues do not fester and become even more widespread. The administrators did not seem to be aware of the extent of the cyberbullying problems on their campuses, nor of the more serious negative impacts that were being experienced by the victims. This gap between what administrators know and what is being experienced in the life worlds of the students and faculty is akin to what has been found in public schools and reported in the K-12 cyberbullying literature [1,16,19].

The findings from this analysis point to the need for university personnel at all levels to better understand and more effectively address the negative online interactions that are occurring on their campuses among students, between students and faculty, and among faculty members. Cyberbullying as affecting people of any age. The impacts of cyberbullying seem to cross age differences as well as status and position differences.

Just as it is important in the K-12 context to address the problems of cyberbullying in an informed way, so it is important to develop research-based policies and programs at the post-secondary level that can help prevent and/or curtail cyberbullying, and also provide victims with clear avenues of reporting, as well as offer support and counselling when harm has occurred. Currently too many students and faculty are suffering the impacts of cyberbullying in isolation, frustrated with their attempts to solve their situations, without any clear guidelines to follow, and within the context of a university culture which benignly seems to tolerate such actions.

## Appendix A

Wording of open-ended survey questions

Student survey:

#59. If you feel comfortable, please give an example of a time when you were cyber-bullied by another student(s) or faculty member, how it made you feel, what you tried to do to stop it, and what happened (please do NOT disclose your identity or that of third parties, e.g., the bully, in your response).

Faculty survey:

#57. If you feel comfortable, please give an example of a time when you were cyber-bullied by a student or students, describing how it made you feel, what you tried to do to stop it, and what happened (please do NOT disclose your identity or that of third parties, e.g., the bully, in your response).

#92. If you feel comfortable, please give an example of a time when you were cyber-bullied by a university colleague, describing how it made you feel, what you tried to do to stop it, and what happened (please do NOT disclose your identity or that of third parties, e.g., the bully, in your response).

## References

[1] Cassidy W., Faucher C., Jackson M. (2013). Cyberbullying among youth: A comprehensive review of current international research and its implications and application to policy and practice. Sch. Psychol. Int..

[2] Beran T.N., Rinaldi C., Bickham D.S., Rich M. (2012). Evidence for the need to support adolescents dealing with harassment and cyber-harassment: Prevalence, progression, and impact. Sch. Psychol. Int..

[3] Cowie H., Bauman S., Coyne I., Myers C., Pörhölä M., Almeida A., Smith P.K., Steffgen G. (2013). Cyberbullying amongst university students: An emergent cause for concern?. Cyberbullying through the New Media: Findings from an International Network.

[4] Cowie H., Myers C.-A., Cowie H., Myers C.-A. (2016). What we know to date about bullying and cyberbullying among university students. Bullying among University Students: Cross-National Perspectives.

[5] Faucher C., Cassidy W., Jackson M. (2015). From the sandbox to the inbox: Comparing the acts, impacts, and solutions of bullying in K-12, higher education, and the workplace. J. Educ. Train. Stud..

[6] Pörhöla M., Cowie H., Myers C.-A. (2016). Do the roles of bully and victim remain stable from school to university? Theoretical considerations. Bullying among University Students: Cross-National Perspectives.

[7] Campbell M., Cowie H., Myers C.-A. (2016). Policies and procedures to address bullying at Australian universities. Bullying among University Students: Cross-National Perspectives.

[8] Faucher C., Jackson M., Cassidy W. (2014). Cyberbullying among university students: Gendered experiences, impacts, and perspectives. Educ. Res. Int..

[9] Cassidy W., Faucher C., Jackson M. (2014). The dark side of the ivory tower: Cyberbullying of university faculty and teaching personnel. Alberta J. Educ. Res..

[10] Grellhesl M., Punyanunt-Carter N.M. (2012). Using the uses and gratifications theory to understand gratifications sought through text messaging practices of male and female undergraduate students. Comput. Hum. Behav..

[11] Wildermuth S., Davis C.B., Wankel L.A., Wankel C. (2012). Flaming the faculty: Exploring root causes, consequences, and potential remedies to the problem of instructor-focused uncivil online student discourse in higher education. Misbehavior Online in Higher Education: Cutting-Edge Technologies in Higher Education.

[12] Blizard L.M. (2016). Faculty members’ experiences of cyberbullying by students at one Canadian university: Impact and recommendations. Int. Res. High. Educ..

[13] McKay R., Arnold D.H., Fratzl J., Thomas R. (2008). Workplace bullying in academia: A Canadian study. Employ. Respons. Rights J..

[14] Vance J.W. (2010). Cyber-Harassment in Higher Education: Online Learning Environments.

[15] Agatston P., Kowalski R., Limber S., Patchin J.W., Hinduja S. (2012). Youth views on cyberbullying. Cyberbullying Prevention and Response: Expert Perspectives.

[16] Cassidy W., Jackson M., Brown K. (2009). Sticks and stones can break my bones, but how can pixels hurt me?. Sch. Psychol. Int..

[17] Hinduja S., Patchin J.W. (2007). Offline consequences of online victimization. J. Sch. Violence.

[18] Hinduja S., Patchin J.W. (2008). Bullying beyond the Schoolyard: Preventing and Responding to Cyberbullying.

[19] Jackson M., Cassidy W., Brown K. (2009). Out of the mouth of babes: Students voice their opinions on cyber-bullying. Long Island Educ. Rev..

[20] Kowalski R.M., Limber S.P., Agatston P.W. (2012). Cyberbullying: Bullying in the Digital Age.

[21] Marczak M., Coyne I. (2010). Cyberbullying at school: Good practice and legal aspects in the United Kingdom. Aust. J. Guid. Couns..

[22] Menesini E., Nocentini A., Costabile A., Spears B.A. (2012). Peer education intervention: Face-to-face versus online. The Impact of Technology on Relationships in Educational Settings.

[23] Nocentini A., Calmaestra J., Schultze-Krumboltz A., Scheithauer H., Ortega R., Menesini E. (2010). Cyberbullying: Labels, behaviours and definition in three European countries. Aust. J. Guid. Couns..

[24] Olweus D. (2012). Cyberbullying: An overrated phenomenon?. Eur. J. Dev. Psychol..

[25] Patchin J.W., Hinduja S., Patchin J.W., Hinduja S. (2012). Cyberbullying: An update and synthesis of the research. Cyberbullying Prevention and Response: Expert Perspectives.

[26] Smith P.K., Jimerson S.R., Nickerson A.B., Mayer M.J., Furlong M.J. (2012). Cyberbullying and cyber aggression. Handbook of School Violence and School Safety: International Research and Practice.

[27] Sourander A., Klomek A.B., Ikonen M., Lindroos J., Luntamo T., Koskelainen M., Ristkari T., Helenius H. (2010). Psychosocial risk factors associated with cyberbullying among adolescents: A population-based study. Arch. Gen. Psychiatry.

[28] Tokunaga R.S. (2010). Following you home from school: A critical review and synthesis of research on cyberbullying victimization. Comput. Hum. Behav..

[29] Von Marées N., Petermann F. (2012). Cyberbullying: An increasing challenge for schools. Sch. Psychol. Int..

[30] Ybarra M.L., Diener-West M., Leaf P.J. (2007). Examining the overlap in internet harassment and school bullying: Implications for school intervention. J. Adolesc. Health.

[31] Arıcak O.T., Cowie H., Myers C.-A. (2016). The relationship between mental health and bullying. Bullying among University Students: Cross-National Perspectives.

[32] Giovazolias T., Malikiosi-Loizos M., Cowie H., Myers C.-A. (2016). Bullying at Greek universities: An empirical study. Bullying among University Students: Cross-National Perspectives.

[33] Kokkinos C.M., Antoniadou N., Markos A. (2014). Cyber-bullying: An investigation of the psychological profile of university student participants. J. Appl. Dev. Psychol..

[34] Rivers I., Cowie H., Myers C.-A. (2016). Homophobic and transphobic bullying in universities. Bullying among University Students: Cross-National Perspectives.

[35] Cassidy W., Jackson M., Faucher C., Navarro R., Yubero S., Larrañaga E. (2016). Gender differences and cyberbullying towards faculty members in higher education. Cyberbullying across the Globe: Gender, Family, and Mental Health.

[36] Taylor S., Lester J. (2012). Workplace bullying: Does tenure change anything? The example of a Midwestern research university. Workplace Bullying in Higher Education.

[37] Vaughan S., Tehrani N. (2012). Ya’makasi or the art of displacement in the corporate world: A target’s perspective on the impact of workplace bullying. Workplace Bullying: Symptoms and Solutions.

[38] Hollis L.P. (2012). Bully in the Ivory Tower: How Aggression and Incivility Erode American Higher Education.

[39] Lampman C. (2012). Women faculty at risk: U.S. professors report on their experiences with student incivility, bullying, aggression, and sexual attention. NASPA J. Women High Educ..

[40] Minor M.A., Smith G.S., Brashen H. (2013). Cyberbullying in higher education. J. Educ. Res. Pract..

[41] Olweus D. (1993). Bullying at School: What We Know and What We Can Do.

[42] Pence E., Paymar M. (1993). Education Groups for Men Who Batter: The Duluth Model.

[43] Campbell M.A. (2005). Cyber bullying: An old problem in a new guise?. Aust. J. Guid. Couns..

[44] DeSouza E.R. (2011). Frequency rates and correlates of contrapower harassment in higher education. J. Interpers. Violence.

[45] Hinduja S., Patchin J.W. (2012). School Climate 2.0: Preventing Cyberbullying and Sexting One Classroom at a Time.

[46] Sallee M., Diaz C., Lester J. (2012). Sexual harassment, racist jokes, and homophobic slurs: When bullies target identity groups. Workplace Bullying in Higher Education.

[47] Crookston R.K. (2012). Working with Problem Faculty: A 6-Step Guide for Department Chairs.

[48] Shariff S., DeMartini A., Cowie H., Myers C.-A. (2016). Cyberbullying and rape culture in universities: Defining the legal lines between fun and intentional harm. Bullying among University Students: Cross-National Perspectives.

[49] Glaser G.B., Strauss A.L. (1967). The Discovery of Grounded Theory: Strategies for Qualitative Research.

[50] Merriam S.B., Tisdell E.J. (2009). Qualitative Research: A Guide to Design and Implementation.

[51] Miles M.B., Huberman A.M., Saldaña J. (1994). Qualitative Data Analysis: A Methods Sourcebook.

